# 

**Published:** 1876-03

**Authors:** 


### TABLE OF CONTENTS.

ORIGINAL COMMUNICATIONS—

Medical College of Georgia. Addresses by Rev. C. A. Evans and Dr. Wm. Arnold Adams ........ 129

Pelvic Examinations by the Rectum. By G. H. Baxter, of Tennessee ........ 147

SELECTIONS—

Chronic Follicular Pharyngitis. By Bernard Tauber, M. D., Cincinnati, Ohio ........ 151
Mammary Disease During Lactation. By Dr. Salvo Caro ........ 154
Case of Chronic Dysentery ........ 157
Hints on the Treatment of Constipation ........ 159

EXTRACTS AND GLEANINGS—

Old friends and New Faces, 161. The Use of Baths in the Summer Complaint of Children, 163. The Puerperal Diseases of the Mammæ, 165. Carbuncle and Felon, 166. Peruvian Bark in Sore Throat, 168. Pruritus, 168. Treatment of Certain Tumors by Subcutaneous Injectiou of Alcohol, 168. Treatment of Anal Vegetations, 169. Ipecac in Dysentery, 170. Properties of Grindelia Robusta, 171. The Hypodermic Use of Apomorphia as an Emetic in Children, 171. Chloral Suppositories, 172. Pernicious Anæmia, 173. Cerebro-Spinal Fever, 173. Method of Instantaneously Arresting Palpitatons of the Heart, 174. Ergot of Rye as an Antipyretic, 174. Purpura Hæmorrhagica, 174. Treatment of Chronic Ulcers of the Leg, 175. Summer Complaint, 175. Tincture of Chloride of Iron for Nasal Polypus, 175. Electricity in Jaundice, 175. Tepid Baths in Fevers, 176. Mercurial Ointment in Boils and Carbuncles, 176. A Cure for Bright's Disease, 176. Vegetable Acids, 176. Treatment of Chronic Constipation, 177. Sexual Hyperchondriasis, 178. Dilatation of the Servix Uteri in Dysmenarrhœa, 179. A New Medicine, 180. Cod Liver Oil and Lacto-Phosphate of Lime, 181. Improved Formula for Camphor Water, 182. An Instrument for the Removal of Retained Placenta, 182. Treatment of Hemiplegia by Large Doses of Bromide of Ammonia, 183. The Theapy Leucocythæmia, 183. Citric Acid in the Treatment of Cancer, 184. Removal of Foreign Bodies from the Ear, 184. Freckle Lotion, 184. Treatment of Itch, 184. Pruritus Treated with Vinegar, 184. Chloral in Nocturnal Emesis, 184.

EDITORIAL AND MISCELLANEOUS—

Kind Words ........ 186
An Advertising Department ........ 188
Our Advertising Department ........ 189
Communications just Received ........ 191

